# Editorial for Special Issue: The Insecticidal Bacterial Toxins in Modern Agriculture

**DOI:** 10.3390/toxins9120396

**Published:** 2017-12-09

**Authors:** Juan Ferré, Baltasar Escriche

**Affiliations:** ERI de Biotecnología y Biomedicina (BIOTECMED), Department of Genetics, University of Valencia, Burjassot, 46100 Valencia, Spain

Agriculture has suffered enormous changes since the first human attempts to domesticate plants to obtain productive varieties which could become a constant source of food. Many developments have shaped current agricultural systems, especially those that led to extensive industrial monocultures. Concurrently with those, there are numerous other types of small scale agricultural systems with an important social and economic impact.

The development of ecosystems with scarce plant varieties has favored the presence of specialized phytophagous that have evolved and adapted to plant species used in agriculture. Pest species share some biological traits, such as short generation cycles and large offsprings. Improvements in agriculture have led to a high production efficiency and the control of pests through different strategies. Modern agriculture seeks to evolve to more environmentally-friendly systems with little environmental impact and accessible to developing countries.

The strategy of pest control based on the use of specific pathogenic microorganisms was already developed at the beginning of the XX century, though its extended commercial use was not achieved until relatively recently, with public awareness of the problems caused by the use of chemical synthetic insecticides.

One of the most successful agents, because of its environmental friendly properties, is the pesticides based on the Gram-positive bacterium *Bacillus thuringiensis*. This bacterium produces protein inclusions during sporulation, known as crystals, which are toxic to some insects and nematodes. The proteins in the parasporal crystal are called Cry proteins, or Bt toxins, and are the active ingredient of *B. thuringiensis* based insecticides. Each Cry protein has a very specific spectrum of action against a few insect species and, therefore, each one is suitable for the control of a determined number of pest insects. Several of these proteins have been expressed in different plants of commercial interest (the so called Bt crops), which become protected from the target pests. The current challenge for Bt crops is to maintain their success with strategies aimed at increasing their efficacy and broadening their spectrum of action while, at the same time, preventing the evolution of resistant populations.

Most of the Bt toxins used so far belong to the so called three-domain toxins (3D-toxins), because they have three well differentiated domains in their structure. The mode of action of these proteins has been studied for a long time, but all the steps involved are not yet fully understood. A key step leading to their specificity is the binding to receptors in the midgut of the target insects. Changes in these receptors may lead to insect resistance to all Cry proteins that share them as binding targets, conferring cross-resistance. Thus, the study of other toxins present in this bacterial species, such as those produced in the vegetative phase (called Cry1I and Vip3), is of interest to complement or replace, in the future, those that are currently in widespread use.

Lepidoptera species control has been a main target for these proteins, but other types of insects, such as Coleoptera, are drawing more attention because of their strong economic impact. In fact, several studies with Bt proteins toxic to Coleoptera are being carried out with 3D-toxins (i.e., Cry3), with dual toxic proteins in Lepidoptera/Coleoptera, (i.e., Cry1I) and with novel binary proteins (i.e., Cry34/Cry35). In these cases, studies on beneficial insects are even more urgent due to the abundant non-pest Coleopteran species that occur in soil ecosystems.

In the following articles, you can find a brief synopsis of a review and ten research papers that make up this special issue entitled “The Insecticidal Bacterial Toxins in Modern Agriculture”.

The screening and characterization of new *B. thuringiensis* strains is described by Djenane et al. [1], by characterizing 157 isolates. Most of them (99%) were shown to have antifungal activity with endochitinase and exochitinase genes, whereas only very few isolates (30%) showed antibacterial activity. Several shapes of parasporal crystals were observed within the 50% of isolates harboring *cry1*, *cry2*, or *cry9* genes. Moreover, 70% of isolates contained a *vip3* gene, suggesting that they could have a wide range of entomotoxic activity.

The accumulation of Cry proteins in the parasporal crystal has been reviewed by Adalat et al. [2]. The crystal form is important to the stability of the proteins in adverse conditions and for long duration. Dense protein accumulation in the crystal has been attributed to the C-terminal end of the 135 kDa Cry proteins, but other proteins have different sizes. The authors studied the genomic organization of several Cry proteins, some of the scarcely studied, and the necessity of helper proteins or other factors for crystallization. These data provide the bases for Cry classification on the ground on their requirements for crystallization.

Vip3A toxins are one of the types of secretable proteins from *B. thuringiensis* (they do not accumulate in the crystal) whose mode of action is even much more poorly understood than the best studied Cry proteins. Vip3A proteins are some of the new toxins implemented on transgenic crops, such as cotton and corn. Bel et al. [3] analyzed the Vip3Aa processing by trypsin and midgut juice, showing that the apparent early degradation of this protein at high concentration of proteases observed by SDS-PAGE is an experimental artifact. This study also confirms that the activated Vip3Aa indeed consists of two polypeptides; one is the N-terminal 20 kDa fragment and the other the 66 kDa C-terminus, which are retained together. Both are extremely resistant to proteases. The data suggested a cluster of beta sheets in the C-terminal region as the basis for the high stability of the 66 kDa core to proteases.

To delay the evolution of resistance to Bt crops it is important that the inheritance of resistance is recessive and that it is associated to fitness costs. Determination of a pest baseline susceptibility and of its resistance genetic basis to a pesticide is key for its implementation and for an early resistance detection. Wang et al. [4] reported data for a Cry1Ie highly resistant (>800-fold) strain of the Asian corn borer, *Ostrinia furnacalis*, based on transgenic maize experiments. The genetic basis was nearly recessive when using maize leaf tissue, but nearly dominant when using maize silk. The resistance was controlled by more than one locus, but it did not conferred cross-resistance to Cry1Ab, Cry1Ac, Cry1F, and Cry1Ah, suggesting that Cry1Ie would be an appropriate candidate for co-expression with other genes currently in use in Bt maize. In other experiments, Wei et al. [5] analyzed Vip3A, one of the new toxins implemented on transgenic cotton, which it is expanding in China before of the use of Cry1Ac and Cry2Aa cotton. They tested the susceptibility of 12 populations of the cotton bollworm *Helicoverpa armigera*, obtaining a 25-fold range of natural variation. The results showed no cross-resistance in four Cry1Ac and Cry2Aa resistant populations indicating that Vip3A is a good alternative for the Cry proteins. In another paper, Paolino and Gassmann [6] analyzed two strains of the coleopteran *Diabrotica virgifera virgifera* (Western Corn Rootworm, WCR) with field-evolved resistance to Cry3Bb1 maize. Using plant-based and diet-based bioassays, the authors revealed that the inheritance of resistance was non-recessive and had no fitness costs associated. These findings highlight the potential for the rapid evolution of resistance to Cry3Bb1 and indicate the necessity of improvement of resistance management strategies for this pest.

Non-3D Cry proteins are still far from having a deep understanding of their mode of action. In fact, only few papers have dealt with the binary toxin Cry34Ab/Cry35Ab, already implemented in transgenic plants to control the WCR. Bowling et al. [7] studied its specific effects on cells and tissues using high-resolution resin-based histopathology methods. In addition, the effects of other toxins—such as Cry3Aa1, Cry6Aa1, and the *Photorhabdus* toxin complex protein TcdA—were documented. Clear symptoms of intoxication were observed for all insecticidal proteins tested, including swelling and sloughing of enterocytes, constriction of midgut circular muscles, stem cell activation, and obstruction of the midgut lumen. On the other hand, Wang et al. [8] analyzed by RNAseq the changes in gene expression profiles after ingestion of the Cry34Ab/Cry35Ab and their isolated components and the group. Most of the genes that showed differences in expression have no significant hits in the NCBI nr database, but some of them were associated with binding and catalytic activity.

The study of the mechanisms of resistance are key to avoid or delay resistance. Pauchet et al. [9] reported data about the poplar beetle pest, *Chrysomela tremula*, with a population resistant to Cry3Aa. The resistance was controlled by a single autosomal locus, and the transcriptome data and cell analyses pointed to a gene from the ABC transporter family as a candidate resistance gene, and its protein as a receptor candidate. This result will strengthen the involvement of ABC proteins in the mode of action for 3D Cry proteins, as it has been described in other insects by other authors.

Minimizing detrimental effects of Bt toxins on non-target organisms is a key issue for their safe use in Bt sprays and Bt crops. This fact is more relevant for proteins that can affect a wider number of insect species, such as Cry1I, which is toxic to Lepidoptera and Coleoptera. Li et al. [10] analyzed the effect of the pure toxin and from Bt-transgenic maize pollen on the predator and pollen feeder, *Propylea japonica*, finding negligible risk for this insect species. In a broader approach, Skoková Habuštová et al. [11] explored the suitability of carabid beetles as surrogates for the detection of unintended effects of genetic modified crops, mainly Bt crops. The study of 86 species showed that a group of just 16 species, representing 15 categories of functional traits, typical dominant inhabitants of agroecocenoses in Central Europe, can be a good indicator of the ecological impact of these crops.

We hope that the novel aspects reported in the present special issue can be of interest to those interested in bacterial toxins, especially those toxins derived from *B. thuringiensis*, in the further research and application in agriculture. This compilation of papers facilitates access to all this information, provides the “state-of-the art” on this topic, and paves the way to future challenges.
